# Randomized Clinical Trial for the Optimization of Dyslipidemia Management in Patients with End-Stage Renal Disease Undergoing In-Hospital Maintenance Hemodialysis Therapy

**DOI:** 10.3390/pharmaceutics17091128

**Published:** 2025-08-28

**Authors:** Sadia Rehman, Muhammad Farhan, Muhammad Raza Sarfraz, Asma Naveed, Fahad Usman, Anila Bibi, Raheel Ahmed, Hiya Huq, Ali Hasan, Jarin Anzoom, Pobitro Kumar

**Affiliations:** 1 Department of Biochemistry, Bahria University of Health Sciences Campus Karachi, Karachi 74400, Pakistan; sadiarehman.bumdc@bahria.edu.pk; 2 Department of Biochemistry, University of Karachi, Karachi 75270, Pakistan; farhankamali@uok.edu.pk; 3 Allied Hospital, Faisalabad Medical University, Faisalabad 38000, Pakistan; mrazasarfraz@outlook.com; 4 Department of Nephrology, PNS Shifa Hospital, Karachi 75530, Pakistan; 5 Department of Community Medicine and Public Health, Sialkot Medical College, Sialkot 51310, Pakistan; 6 Department of Biochemistry, Jinnah Sindh Medical University, Karachi 75510, Pakistan; 7 Department of Medicine, National Heart and Lung Institute, Imperial College London, London SW7 2AZ, UK; ali.hasan21@imperial.ac.uk; 8 Department of Psychology, Health and Professional Development, Oxford Brookes University, Oxford OX3 0BP, UK; 9 School of Allied and Public Health, University of Chester, Chester CH1 4BJ, UK; 10 Department of Medicine, Rangpur Medical College, Rangpur 5400, Bangladesh

**Keywords:** hemodialysis, renal replacement therapy, carnitine, acetylcarnitine, hyperlipidemias

## Abstract

**Background/Objectives**: End-stage renal disease (ESRD) patients on maintenance hemodialysis (MHD) frequently develop L-carnitine (LC) deficiency, leading to dyslipidemia and increased cardiovascular risk. While LC supplementation may improve dyslipidemia, the optimal route of administration remains unclear. This study evaluates the effects of LC on dyslipidemia in MHD patients and compares oral versus intravenous (IV) administration. **Methods**: In this dual-center randomized controlled trial (NCT05817799), 102 MHD patients aged 18–50 years were randomized to receive either oral (500 mg thrice daily) or IV LC (20 mg/kg post-dialysis thrice weekly for 23 weeks followed by 500 mg oral daily for 1 week) for 24 weeks, and blood samples were obtained to evaluate lipid profile parameters. **Results**: Eighty-three patients completed the study (oral *n* = 49, IV *n* = 34). Both groups demonstrated significant improvements in all lipid parameters (*p* < 0.0001). In the oral group, total cholesterol (TC) demonstrated a mean reduction of 15.04 ± 8.52, triglycerides (TG) decreased by 14.84 ± 13.20, and low-density lipoprotein cholesterol (LDL-C) declined by 9.87 ± 8.74, with a rise in high-density lipoprotein (HDL) of 5.34 ± 4.33. In contrast, the IV group showed greater improvement, with TC being reduced by 17.62 ± 8.98, TG reduced by 19.21 ± 11.33, and HDL-C elevated by 7.26 ± 4.35. Group comparison revealed significantly greater LDL reduction in the IV group (71.91 ± 14.37 mg/dL) versus oral group (79.04 ± 14.92 mg/dL, *p* = 0.03), whereas TC, TG, and HDL changes showed no significant differences (*p* > 0.05). **Conclusions**: Both oral and IV interventions effectively improved lipid profiles, and IV administration showed a more pronounced effect on LDL reduction, suggesting potentially greater efficacy of IV administration for LDL reduction.

## 1. Introduction

Patients undergoing maintenance hemodialysis (MHD) face a markedly increased risk of cardiovascular disease (CVD), in which dyslipidemia, characterized by abnormal levels of total cholesterol (TC), triglycerides (TGs), low-density lipoprotein cholesterol (LDL), and high-density lipoprotein cholesterol (HDL), is a major and modifiable contributor [1]. These abnormalities accelerate atherogenesis and intensify the cardiovascular complications inherent to chronic kidney disease (CKD) and MHD [2], while elevated lipid levels further increase the likelihood of adverse cardiovascular outcomes [3].

**Figure 1:**
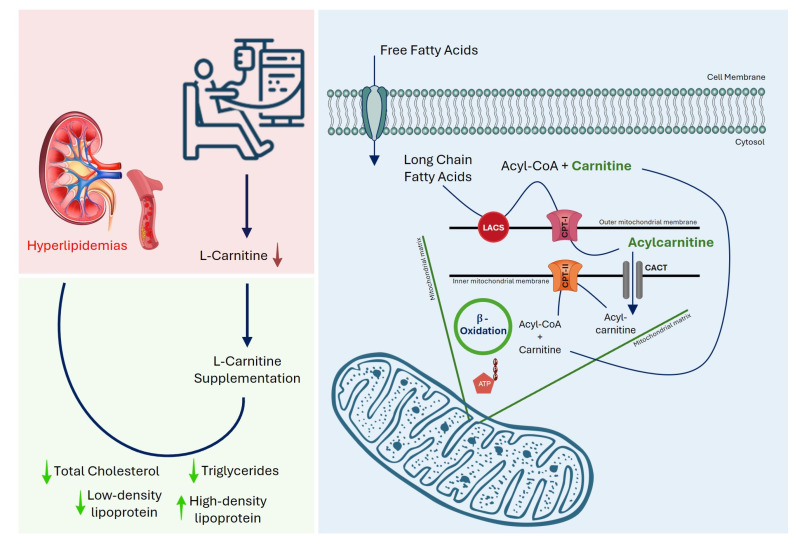
Mechanism of L-carnitine deficiency in maintenance hemodialysis patients and its impact on lipid metabolism. Chronic kidney disease and hemodialysis are associated with reduced circulating L-carnitine levels, which impair mitochondrial β-oxidation of long-chain fatty acids, leading to dyslipidemia. L-carnitine facilitates the transport of long-chain fatty acids into the mitochondrial matrix via conversion to acylcarnitine and subsequent β-oxidation, thereby improving lipid parameters. Total cholesterol (TC), triglycerides (TGs), low-density lipoprotein cholesterol (LDL), high-density lipoprotein cholesterol (HDL), long-chain acyl-CoA synthetase (LACS), carnitine palmitoyltransferase (CPT), carnitine-acylcarnitine translocase (CACT), adenosine triphosphate (ATP).

Multiple factors influence dyslipidemia in MHD patients, including the severity of renal dysfunction, dialysis duration, nutritional status, inflammation, and carnitine deficiency [1]. CKD disrupts carnitine balance through reduced endogenous synthesis and increased losses during dialysis. Dialysis-related carnitine deficiency is associated with erythropoietin-resistant anemia, intradialytic hypotension, muscle cramps, reduced exercise tolerance, cardiovascular instability, and skeletal muscle weakness [4]. Carnitine, a water-soluble quaternary amine, is essential for mitochondrial beta-oxidation of long-chain fatty acids, facilitating their transport across the inner mitochondrial membrane for energy production (Figure 1). Deficiency impairs this process, leading to lipid accumulation in circulation, while reduced hepatic and lipoprotein lipase activity in MHD patients further worsens lipid metabolism [5]. Since TC and TGs are key indicators of serum lipid status, their optimal control is crucial for reducing cardiovascular morbidity and mortality in this population [6]. Carnitine also regulates free fatty acid (FFA) availability for triglyceride synthesis, which may improve lipid profiles in deficient patients [7].

Evidence links carnitine deficiency to uremic hyperlipidemia [8], and free carnitine levels show a negative correlation with TG concentrations in MHD patients [9]. Previous studies have reported that serum carnitine levels may fall to 40% of baseline during a single dialysis session [10]. Experimental and clinical studies indicate that L-carnitine (LC) supplementation can improve lipid metabolism. Animal studies show normalization of TC and TG levels [11] and prevention of atherosclerosis progression through antioxidant and lipid-lowering effects [12]. However, findings from randomized controlled trials (RCTs) in humans are inconsistent, with some demonstrating lipid improvements and others showing no significant benefit [13,14], likely due to differences in population characteristics, diet, genetics, sample sizes, and study criteria [15,16].

Given that LC deficiency is prevalent among MHD patients and may contribute to adverse lipid profiles and considering the inconsistent findings from prior studies as well as the scarcity of data from South Asian populations, this study evaluates and compares the effects of oral and intravenous LC supplementation on lipid profiles in Pakistani MHD patients to provide evidence for practical, cost-effective, and regionally relevant treatment strategies.

## 2. Materials and Methods

### 2.1. Setting and Participants

A dual-center randomized controlled trial was conducted at two hemodialysis units in Karachi, Pakistan, from January to July 2023, after obtaining ethical approval from the Institutional Ethics Review Committee of Jinnah Postgraduate Medical Centre, Karachi, Pakistan, (Ref. No. F.2-81/2020-GENL/42532/JPMC), and Pakistan Navy Station Shifa Hospital, Karachi, Pakistan, (Ref. No. ERC/2022/MEDICINE/23). The study was registered at ClinicalTrials.gov under registration number NCT05817799. Patients were thoroughly informed regarding the study’s purpose, procedures, benefits, and risks. Voluntary written and understood consent was obtained from all participants. OpenEpi Version 3.01 (Dean AG et al., Rollins School of Public Health, Emory University, USA; www.openepi.com, accessed on 9 September 2022) was used to calculate the sample size in line with the existing literature [17]. A minimum of 70 participants was required based on the sample size calculation; to offset possible losses to follow-up, we enrolled 102 individuals.

### 2.2. Inclusion and Exclusion Criteria

The study participants were required to meet the following conditions: (1) a confirmed diagnosis of ESRD with at least two years of continuous MHD therapy (three sessions per week), (2) age between 18 and 50 years, (3) clinical stability, defined as no recent acute complications or hospitalizations within the past three months, (4) ability to provide informed, written, and voluntary consent, and (5) demonstrated willingness and capacity to adhere to the study protocol, including supplementation requirements. Participants were excluded based on the following criteria: (1) patients currently or recently (within the past three months) using lipid-lowering medications, such as statins or fibrates, (2) presence of metabolic or endocrine disorders, including thyroid dysfunction, (3) chronic or acute liver disease, active infections, recent blood transfusions, or other inflammatory conditions, (4) known hypersensitivity to LC or its formulations, (5) prior LC supplementation within the last six months, (6) pregnancy or lactation, (7) diagnosed psychiatric illness or history of substance abuse, and (8) concurrent use of immunosuppressants, antibiotics, or steroids.

### 2.3. Randomization

Eligible patients were allocated to either the Oral or IV group through a 1:1 randomized sequence. Randomization was overseen by an independent biostatistician using a computer-generated block randomization method. Allocation concealment was ensured by sealed opaque envelopes, managed by a study coordinator (a registered nurse with specialized training in clinical research protocols).

### 2.4. Interventions

Dosages for both oral and IV treatments were selected based on established guidelines and previous research [17]. The Oral group received 500 mg LC tablets, taken orally three times daily for 24 weeks under healthcare provider supervision. The IV group received LC supplementation intravenously at 20 mg/kg body weight after each hemodialysis session three times a week for 23 weeks, followed by a maintenance oral dose of 500 mg daily for one additional week. In the final week of the intervention, patients in the intravenous administration group were transitioned to an oral maintenance dose of LC (500 mg daily). This strategy was employed to provide a gradual transition and avoid abrupt discontinuation of supplementation, thereby minimizing potential physiological disruptions and ensuring the stability of therapeutic carnitine levels as the study concluded.

The hemodialysis procedure was performed using Fresenius 4008S dialysis machine (Fresenius Medical Care AG & Co. KGaA, Bad Homburg, Germany; Serial No. 4008S-0987654) equipped with Fresenius Polysulfone^®^ F8 HPS dialyzers (Batch No. F8HPS-2024001, Fresenius Medical Care, Bad Homburg, Germany). Hemodialysis was performed using dialyzers equipped with synthetic membranes, maintaining a blood flow rate of 200–240 mL/min and a dialysate flow rate of 500 mL/min. The equipment used adhered to international safety and performance standards, with all units regularly maintained and calibrated according to the manufacturer’s recommendations. Specific equipment brands and batch numbers were not documented as part of the study protocol, but standard devices widely used in clinical practice were employed. At the baseline and end of the 24th week, 5 mL of venous blood was collected using heparinized, gel-coated vacutainer tubes, for all the patients. All collected samples were centrifuged at 3000 rpm for 10 min, and the separated components were stored at −20 °C for subsequent analysis. Biochemical parameters, including TC, TGs, HDL, and LDL cholesterol levels were measured using the colorimetric method.

### 2.5. Monitoring and Follow-Up

Regular physical examinations were conducted during dialysis sessions, with vital signs monitored daily, particularly in the Oral and IV groups. Patients in the Oral and IV groups were administered LC supplements through different routes of administration to evaluate the effectiveness of both methods in improving patients’ lipid profiles. The primary outcomes were improvement in the lipid profile parameters of the patients in the Oral and IV group and comparison of the efficacy of the oral versus intravenous administration of LC supplements.

### 2.6. Statistical Analysis

Statistical analyses were performed using SPSS version 23.0 (IBM Corporation, Armonk, NY, USA) and GraphPad Prism version 9.0.0 (121) (GraphPad Software Inc., San Diego, CA, USA). The Shapiro–Wilk test was employed to assess the normality of data distribution, and a further statistics test was carefully chosen according to the normal distribution of the data. Continuous variables from baseline and follow-up were expressed as mean  ±  standard deviation (SD), while categorical variables were presented as frequencies and percentages. Differences between groups for categorical variables were assessed using the chi-square test. For continuous variables, paired samples *t*-tests were used to compare pre- and post-intervention differences within each group, whereas unpaired samples *t*-tests were applied to compare baseline and post-intervention variables between the Oral and IV groups to evaluate the relative efficacy of each route of administration. A *p*-value  <  0.05 (two-tailed) was considered statistically significant.

## 3. Results

### 3.1. Study Population and Demographics

**Figure 2:**
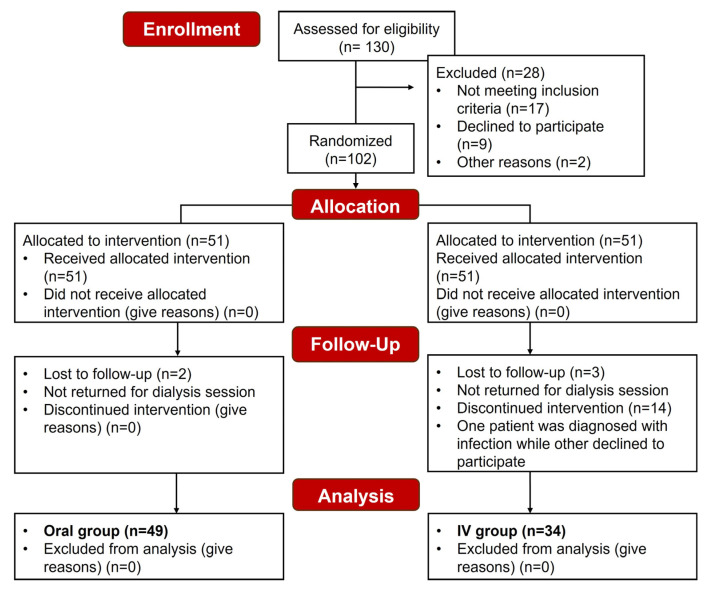
Consolidated Standards for Reporting Trials (CONSORT) diagram showing the subject recruitment and outcome measurement workflow.

A total of 83 patients were included in the final analysis, 49 in the Oral group and 34 in the IV group (Figure 2).

The baseline data provided in our previous study demonstrates a relatively balanced distribution of participants’ characteristics, including age, gender, BMI, and comorbidities, across the Oral and IV groups [18]. The overall mean age of the participants in the Oral group was 44.1 ± 8.92, and 45.62 ± 8.61 in the IV group (*p* = 0.34); of these 83 participants, 41 were male and 42 were female. The mean values obtained for BMI were 25.51 ± 4.69 and 24.55 ± 5.09 in the Oral and IV group, respectively (*p* = 0.34). Female patients were more populous than males in the IV group; however, the gender distribution across both groups showed non-significant difference (*p* = 0.89) (Table 1). Furthermore, in our pilot analysis, we observed a significant deficiency of carnitine levels in MHD patients, with plasma acylcarnitine (AC) concentrations of 22.8 ± 3.6 nmol/L and plasma free carnitine (FC) concentrations of 17.6 ± 4.7 µmol/L, compared with the control group, which had AC levels of 23.57 ± 4.1 nmol/L and FC levels of 55.32 ± 7.3 µmol/L [19].

### 3.2. Changes in Lipid Profile Parameters in the Oral Group

**Figure 3:**
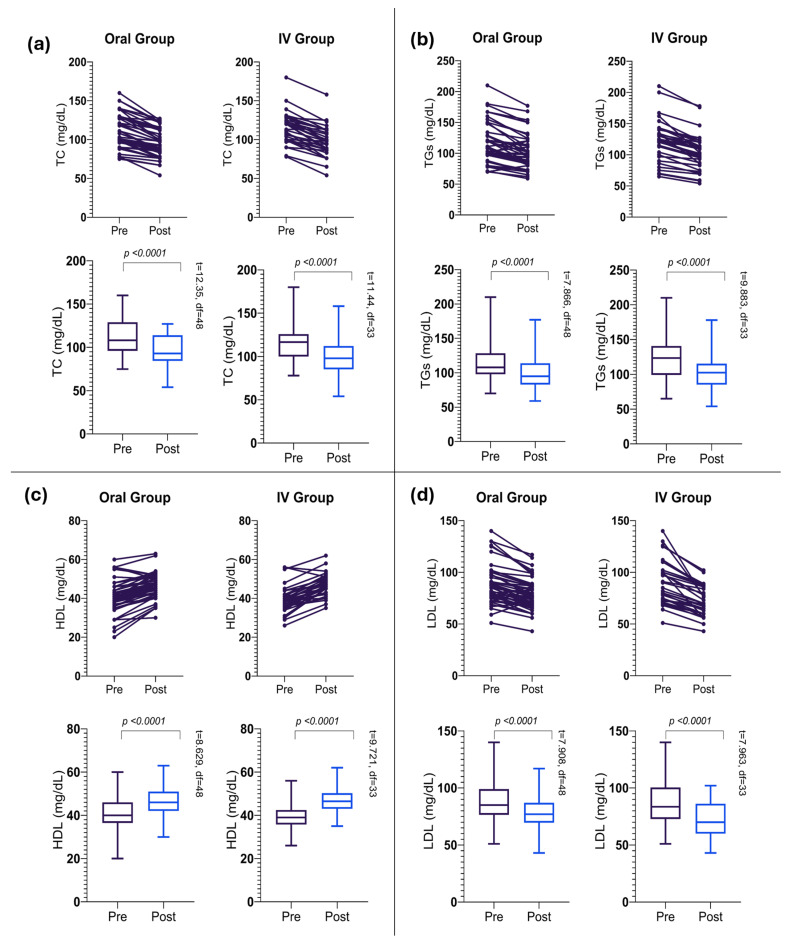
Overall trends in lipid profile improvement. (**a**) Reduction in total cholesterol (TC), (**b**) triglycerides (TGs), (**c**) high-density lipoprotein cholesterol (HDL), (**d**) low-density lipoprotein cholesterol (LDL) following 24-week intervention across all participants.

**Figure 4:**
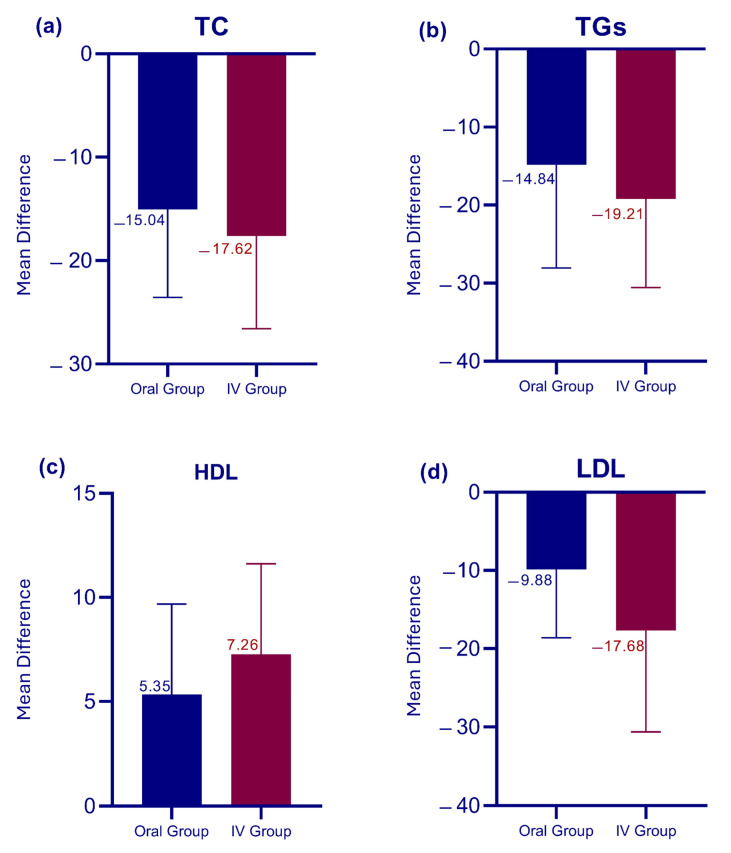
Comparative effects of oral versus intravenous therapy on lipid profile. (**a**) Changes in total cholesterol (TC), (**b**) triglycerides (TGs), (**c**) high-density lipoprotein (HDL), and (**d**) low-density lipoprotein (LDL).

The oral intervention resulted in significant changes across all lipid profile parameters after 24 weeks of treatment (Table 2). TC levels showed a significant reduction from 111.33 ± 21.92 mg/dL to 96.29 ± 18.11 mg/dL (*p* < 0.0001), as shown in (Figure 3a), with a mean decrease of 15.04 ± 8.52 mg/dL (as depicted in Figure 4a). TG levels followed a similar trend, decreasing significantly to 100.55 ± 27.01 mg/dL from their baseline value (as shown in Figure 3b), with a mean reduction of 14.84 ± 13.20 mg/dL (*p* < 0.0001), as depicted in (Figure 4b).

Notably, HDL cholesterol levels showed a significant increase (as shown in Table 2 and Figure 3c) with a mean increase of 5.34 ± 4.33 mg/dL (*p* < 0.0001), as depicted in (Figure 4c). However, the LDL cholesterol levels decreased significantly (as shown in Table 2) with a mean reduction of 9.87 ± 8.74 mg/dL (*p* < 0.0001). The overall reduction in LDL levels after the intervention is indicated in Figure 4d.

### 3.3. Changes in Lipid Profile Parameters in the IV Group

The IV intervention group demonstrated significant changes in all lipid parameters over the 24-week treatment period (Table 3). TC levels showed a marked reduction to 96.85 ± 19.82 mg/dL, with a mean decrease of 17.62 ± 8.98 mg/dL (*p* < 0.0001), as shown in (Figure 3a). The IV group exhibited a slightly larger mean decrease compared to the oral group (as shown in Figure 4a). TG levels decreased substantially to 103.32 ± 28.61 mg/dL from their baseline levels (as shown in Table 2), with a mean reduction of 19.21 ± 11.33 mg/dL (*p* < 0.0001), as depicted in (Figure 3b). HDL cholesterol levels increased overall, with a mean increase of 7.26 ± 4.35 mg/dL (*p* < 0.0001). The IV group demonstrated a larger mean increase in HDL levels compared to the Oral group (as shown in Figure 3c and Figure 4c). However, LDL cholesterol exhibited a considerable reduction in their mean levels post-intervention (Table 2).

### 3.4. Intervention Outcomes and Group Comparison

Overall, the results of our intervention are highly significant (as shown in Table 2 and Table 3) with a *p*-value of <0.0001 for all parameters across both groups. Notably, the most pronounced effect is observed in LDL levels between the Oral and IV groups. Figure 3 illustrates the overall trends highlighting a reduction in TC, TGs, and LDL levels, alongside an increase in HDL levels among all participants. Figure 4 provides a comparative analysis between the IV and Oral groups, revealing that participants in the IV group exhibited a remarkable improvement across all parameters compared to the Oral group. The IV group consistently showed larger mean changes, particularly in LDL reduction and HDL elevation. Table 4 presents the results of the unpaired *t*-test, indicating that pre-intervention levels of all parameters showed no significant differences between the two groups (all *p* > 0.05). Post-intervention analysis revealed similar effects on TC, TGs, and HDL levels between groups (all *p* > 0.05); however, a significantly greater LDL reduction was observed in the IV group (71.91 ± 14.37 mg/dL) compared to the Oral group (79.04 ± 14.92 mg/dL) with *p* = 0.03. These findings suggest that while both administration routes are equally effective in modulating TC, TGs, and HDL levels, the IV route may offer a more substantial benefit in reducing LDL levels.

## 4. Discussion

Our results show significant improvements in lipid parameters in both groups, aligning with previous studies that reported beneficial effects of LC on lipid metabolism [14,20,21,22,23]. Nevertheless, these changes were statistically significant; their magnitude was modest, warranting careful interpretation in light of conflicting evidence and the practical considerations of clinical application. Disorders of carnitine metabolism are well-documented in uremic patients. Although disorders in carnitine metabolism are well-documented among uremic patients [24], carnitine deficiency remains inadequately characterized within the Pakistani population. Consequently, we conducted a preliminary pilot analysis that revealed significantly reduced carnitine levels among MHD patients in our geographical region [25]. This deficiency can develop progressively during chronic MHD, primarily due to both dialysate losses and reduced endogenous synthesis by damaged kidneys. Notably, LC depletion may occur gradually even when baseline serum levels are comparable to those of healthy controls [13,17]. For instance, pre-dialysis serum carnitine concentrations have been reported to decline by approximately 22% within the first year of hemodialysis in patients receiving a placebo, with deficiency manifesting in about 30% of cases [26].

Despite the promising therapeutic potential of LC supplementation, the evidence base remains characterized by substantial heterogeneity and conflicting outcomes that demand critical examination [22]. While our study established significant improvements in lipid parameters, effect sizes documented throughout the literature vary markedly according to study design, population characteristics, and intervention protocols, thereby necessitating cautious interpretation of findings. Although our results, in conjunction with several supportive investigations [20,21,22,23,27,28], substantiate the beneficial effects of LC supplementation, contradictory findings from other studies [29,30] require careful consideration within this evidence framework. Our study reported clinically meaningful lipid modifications, with the oral group demonstrating mean reductions of 15.04 mg/dL in TC and 14.84 mg/dL in TGs, while the IV group exhibited more pronounced improvements with corresponding decreases of 17.62 mg/dL and 19.20 mg/dL, respectively. Our findings corroborate several contemporaneous investigations reporting similar beneficial effects. Fu et al. demonstrated that three-month IV LC treatment (1 g) significantly reduced both TG and LDL levels, with LDL concentrations declining from 119 ± 21 mg/dL to 98 ± 19 mg/dL [27]. Similarly, Emami Naini et al. conducted a randomized, double-blind, placebo-controlled trial administering 1 g/day LC to 24 ESRD patients over 16 weeks, documenting significant TG reductions (−31.1 ± 38.7 mg/dL) and HDL increases (3.7 ± 2.8 mg/dL), though with non-significant changes in TC and LDL [31]. Notably, while their reported HDL improvement of 3.7 mg/dL was modest compared to our findings of 5.34 mg/dL (oral) and 7.26 mg/dL (IV), both studies consistently demonstrate carnitine’s capacity to enhance HDL metabolism. Furthermore, Singh et al., conducting their investigation in India with geographical proximity to our Pakistani cohort, reported that biweekly post-dialysis IV LC administration (1 g) over six months produced comprehensive lipid profile improvements, including decreased TGs, TC, LDL, and VLDL alongside significant HDL elevation, thereby reinforcing both the clinical efficacy and geographical relevance of our comparable outcomes [28].

Contrary to our findings, several investigations have reported divergent outcomes that underscore the complexity of LC supplementation effects. Weschler et al. examined high-dose LC administration (3 g/day) in ten uremic MHD patients and documented paradoxical outcomes, including exacerbated hypertriglyceridemia and increased platelet aggregation in patients predisposed to thromboembolic events, with TG levels rising from 180 ± 66 mg/dL to 219 ± 88 mg/dL after one month [32]. This suggests that excessive LC dosing may precipitate adverse lipid effects in susceptible populations, highlighting the critical importance of optimal dosing strategies [23]. The dosage and duration of LC supplementation emerge as pivotal determinants of therapeutic efficacy [32]. Our protocol employed an IV regimen (20 mg/kg thrice weekly) followed by oral maintenance therapy (500 mg daily), which aligns with evidence supporting enhanced efficacy through higher doses and prolonged supplementation [17]. This approach contrasts with variable dosing strategies employed in other investigations, which may partially explain the heterogeneous outcomes observed across studies. Furthermore, dose–response relationships with LC supplementation exhibit considerable complexity, influenced by both treatment duration and lipid parameter variability. Emami Naini et al. reported that oral LC at 1 g/day reduced TGs from 165.5 mg/dL at baseline to 147.2 mg/dL and 138.3 mg/dL at 8 and 16 weeks, respectively, while TC increased slightly from 147.1 mg/dL to 154.0 mg/dL and 154.5 mg/dL at the same time points, and HDL rose from 30.2 to 31.0 and 34.0, respectively [31]. However, contradictory findings from Katalinic et al., who administered 1 g intravenously post-dialysis over 12 months in 50 MHD patients, revealed significant TC increases, HDL reductions, and unchanged TGs levels [33], outcomes diametrically opposed to our observations. These discrepancies may reflect dose-dependent carnitine effects, as proposed by several investigators [14,33], or alternatively suggest that patient-specific factors, baseline metabolic status, dietary habits and concurrent therapies significantly modulate treatment responses beyond simple dose considerations.

Both IV and oral LC formulations have received regulatory approval for use in MHD patients [34]. While our comparative analysis demonstrated similar therapeutic outcomes for TC, TGs, and HDL across both administration routes, IV LC yielded significantly superior LDL reduction compared to oral supplementation. Fukami et al. reported that transitioning MHD patients to IV therapy for one month produced significant HDL elevation and a trend toward improved LDL-HDL ratio reduction (from 1.69 ± 0.75 to 1.58 ± 0.78). However, their findings revealed no significant LDL modification, contrasting markedly with our observed substantial LDL reductions [35]. Although our findings suggest IV administration’s potential superiority for LDL management, clinical implementation must be contextualized within real-world practice constraints. Pediatric studies further illustrate the complexity of LC responses across different patient populations. A recent investigation involving children aged 6–18 years on MHD demonstrated that LC supplementation (50 mg/kg/day over 10 weeks) significantly reduced serum LDL and TC concentrations alongside unchanged TGs, HDL, and ApoA1 levels [13]. Conversely, Verrina et al. reported that oral LC administration (20 mg/kg/day) in pediatric peritoneal dialysis patients successfully normalized circulating FC levels despite ongoing dialysate losses yet produced no measurable impact on lipid profiles over three months [36]. These pediatric findings contrast with our adult cohort outcomes, suggesting that age-related metabolic differences, treatment modality variations (hemodialysis versus peritoneal dialysis), and intervention duration disparities may substantially influence therapeutic responses. The extended intervention period employed in our study (compared to the three-month pediatric trial) may partially explain the more pronounced lipid improvements observed in our adult population.

Despite IV administration ensuring complete bioavailability and representing the preferred approach for patients with severe deficiency or malabsorption disorders, routine clinical adoption faces substantial limitations including elevated costs, mandatory in-center administration requirements, heightened infection risks associated with prolonged vascular access, and implementation challenges within resource-constrained healthcare settings. Consequently, the theoretical biochemical advantages of IV LC must be balanced against practical clinical considerations and healthcare system capabilities. Our dosing regimen for both treatment groups was strategically selected based on evidence supporting the efficacy of sustained LC supplementation [17]. However, the considerable variability observed across published protocols indicates that optimal dosing strategies and administration routes for lipid modulation remain inadequately defined within the current literature. Critically, emerging evidence suggests the existence of dose-dependent therapeutic thresholds, with certain investigations documenting paradoxical benefit reversal wherein excessive LC administration may exacerbate TGs levels in metabolically susceptible individuals [31]. While this phenomenon was not observed within our study cohort, these findings underscore the importance of individualized dosing considerations and careful patient monitoring in clinical practice implementation.

Our study demonstrates several key methodological strengths, utilizing an evidence-based dose protocol. Regarding novelty, while oral versus IV LC has been studied separately, very few studies have compared administration routes, and evidence from South Asian ESRD populations remains scarce. To our knowledge, this represents the first randomized trial conducted in Pakistan reporting oral versus IV LC outcomes. Our work considers regional differences and dialysis practices that may influence lipid metabolism, while the standardized protocols and practical dosing regimen enhance both methodological rigor and real-world clinical applicability.

Our study has several important limitations that warrant consideration. The study population was restricted to the Pakistani population, potentially limiting generalizability to diverse ethnic and geographic populations, as cultural, dietary, and genetic factors may substantially influence treatment outcomes. The 24-week intervention period, while adequate for assessing short-term biochemical changes, lacked extended follow-up monitoring to evaluate long-term efficacy, safety profiles, and sustained therapeutic effects. Our exclusion criteria for patients with certain metabolic and medical conditions may restrict applicability to broader hemodialysis populations, particularly those with complex comorbidities commonly encountered in clinical practice. Important confounding variables including dietary habits, physical activity levels, and dialysis adequacy parameters were not systematically recorded, despite their potential influence on lipid metabolism outcomes. The study design focused exclusively on biochemical lipid parameter changes without incorporating mechanistic pathway analysis, cardiovascular risk scoring, or cost-effectiveness evaluations, which would strengthen clinical relevance and are planned for future investigations. Moreover, the relatively modest absolute differences between the Oral and intravenous LC groups suggest that larger, multi-center randomized controlled trials with longer follow-up and comprehensive outcome assessments are warranted to confirm these findings and inform evidence-based clinical recommendations. Future research should address these methodological limitations through multi-ethnic validation studies, broader inclusion criteria, systematic recording of lifestyle factors, mechanistic investigations, and comprehensive clinical outcome assessments to better define LC’s role in ESRD dyslipidemia management.

## 5. Conclusions

This study demonstrates that LC supplementation effectively improves lipid profiles in MHD patients. Both routes of LC administration showed improvements in all lipid profile parameters. However, IV administration demonstrated greater efficacy in reducing LDL levels. Our findings help guide clinical decision-making regarding supplementation route selection, warranting careful interpretation in light of conflicting evidence and requiring consideration of patient affordability alongside therapeutic goals.

## Figures and Tables

**Table 1:** Comparison of patient demographics and baseline characteristics.

Patient Characteristics	Oral Group (*n* = 49)	IV Group (*n* = 34)	*p*-Value
Male, *n* (%)	25 (60.98%)	16 (57.41%)	0.89 ^1^
Female, *n* (%)	24 (39.02%)	18 (42.86)	
Age (years), Mean ± SD	44.1 ± 8.92	45.62 ± 8.61	0.44 ^2^
BMI, Mean ± SD	25.51 ± 4.69	24.55 ± 5.09	0.34 ^2^
Co-morbidities, *n* (%)			
Diabetes	8 (16.3%)	6 (17.6%)	0.25 ^1^
Hypertension	23 (46.9%)	14 (41.2%)	
Cardiovascular disorders	2 (4.1%)	6 (17.6%)	
None	16 (32.7%)	8 (23.5%)	

Note. Body mass index (BMI), frequency (*n*), percentage (%), chi-square test (^1^), unpaired sample *t*-test (^2^).

**Table 2:** Comparison of lipid profile parameters before (pre) and after 24 weeks (post) of intervention in the Oral group.

Variables	Oral Group (*n* = 49)					
	Pre-Intervention	Post-Intervention	Differences in the Value	95% CI	*t*-Value	*p*-Value
	Mean ± SD					
TC, mg/dL	111.33 ± 21.92	96.29 ± 18.11	−15.04 ± 8.52	−17.49 to −12.59	12.35	<0.0001 *
TGs, mg/dL	115.39 ± 31.27	100.55 ± 27.01	−14.84 ± 13.20	−18.63 to −11.04	7.87	<0.0001 *
LDL, mg/dL	88.92 ± 19.00	79.04 ± 14.92	−9.87 ± 8.74	−12.39 to −7.36	7.91	<0.0001 *
HDL, mg/dL	40.90 ± 8.36	46.24 ± 6.45	5.34 ± 4.33	4.11 to 6.59	8.63	<0.0001 *

Note: Total cholesterol (TC), triglycerides (TGs), low-density lipoprotein cholesterol (LDL), high-density lipoprotein cholesterol (HDL). Paired sample *t*-test, 95% confidence interval (CI) of the difference, significant value (*).

**Table 3:** Comparison of lipid profile parameters before (pre) and after 24 weeks (post) of intervention in the IV group.

Variables	Oral Group (*n* = 34)					
	Pre-Intervention	Post-Intervention	Differences in the Value	95% CI	*t*-Value	*p*-Value
	**Mean ± SD**					
TC, mg/dL	114.47 ± 20.12	96.85 ± 19.82	−17.62 ± 8.98	−20.75 to −14.48	11.44	<0.0001 *
TGs, mg/dL	122.53 ± 33.60	103.32 ± 28.61	−19.21 ± 11.33	−23.16 to −15.25	9.88	<0.0001 *
LDL, mg/dL	89.59 ± 21.10	71.91 ± 14.37	−17.68 ± 12.94	−22.19 to −13.16	7.96	<0.0001 *
HDL, mg/dL	39.56 ± 6.95	46.82 ± 5.72	7.26 ± 4.35	5.74 to 8.79	9.72	<0.0001 *

Note: Paired sample *t*-test, 95% confidence interval (CI) of the difference, significant value (*).

**Table 4:** Comparative analysis of lipid profile parameters (pre- and post-intervention) between Oral and intravenous groups.

Test Results	Pre-Intervention				Post-Intervention			
	Oral Group	IV Group	t-Value	*p*-Value	Oral Group	IV Group	*t*-Value	*p*-Value
TC, mg/dL	111.33 ± 21.92	114.47 ± 20.12	−0.66	0.51	96.29 ± 18.11	96.85 ± 19.82	−0.13	0.89
TGs, mg/dL	115.39 ± 31.27	122.53 ± 33.60	−0.99	0.32	100.55 ± 27.01	103.32 ± 28.61	−0.45	0.65
LDL, mg/dL	88.92 ± 19.00	89.59 ± 21.10	−0.16	0.88	79.04 ± 14.92	71.91 ± 14.37	2.19	0.03 *
HDL, mg/dL	40.90 ± 8.36	39.56 ± 6.95	0.80	0.44	46.24 ± 6.45	46.82 ± 5.72	−0.34	0.67

Note: Unpaired sample *t*-test, significant value (*).

## Data Availability

The data presented in this study are available on request from Sadia Rehman (Principal Investigator).
